# Highly Symptomatic Progressing Cardiac Paraganglioma With Intracardiac Extension Treated With ^177^Lu-DOTATATE: A Case Report

**DOI:** 10.3389/fendo.2021.705271

**Published:** 2021-07-22

**Authors:** Alexis Huot Daneault, Mélanie Desaulniers, Jean-Mathieu Beauregard, Alexis Beaulieu, Frédéric Arsenault, Geneviève April, Éric Turcotte, François-Alexandre Buteau

**Affiliations:** ^1^ Département de médecine nucléaire et radiobiologie, Université de Sherbrooke, Sherbrooke, QC, Canada; ^2^ Département d’imagerie médicale, Division médecine nucléaire, CHU de Québec, Québec, QC, Canada

**Keywords:** PRRT, 177Lu-DOTATATE, metastatic cardiac paraganglioma, personalized activity, dosimetry

## Abstract

**Introduction:**

Primary cardiac paragangliomas are rare tumors. Metastatic disease is even rarer. Surgical management is technically challenging, and sometimes even impossible. Available therapeutic modalities for metastatic disease include external beam radiation therapy as well as systemic treatments, namely ^131^I-MIBG and more recently, peptide receptor radionuclide therapy (PRRT) with ^177^Lu-DOTATATE. To our knowledge, this is the first case of progressive unresectable cardiac paraganglioma with intracardiac extension treated with dosimetry based personalized PRRT to be reported. This case is of particular interest since it documents for the first time the efficacy, and especially the safety of the ^177^Lu-DOTATATE PRRT in this precarious context for which therapeutic options are limited.

**Case Presentation:**

A 47-year-old man with no medical history consulted for rapidly decreasing exercise tolerance. The investigation demonstrated an unresectable progressing metastatic cardiac paraganglioma with intracardiac extension. The patient was treated with personalized ^177^Lu-DOTATATE PRRT and showed complete symptomatic and partial anatomical responses, with a progression-free survival of 13 months.

**Conclusions:**

PRRT with ^177^Lu-DOTATATE should be considered for inoperable cardiac paraganglioma. No major hemodynamic complications were experienced. Therapy resulted in safety and substantially improved quality of life.

## Introduction

Paragangliomas (PGLs) are rare neuroendocrine tumors arising from the chromaffin cells of the neural crest (1). Their incidence is estimated to be less than 1 per 100,000 people per year (2). PGLs can be distributed along sympathetic or parasympathetic chains from the base of the skull to the prostate, but the majority are located in the abdomen (3). Cardiac PGLs are extremely rare tumors and account for only 2% of all PGLs (4, 5) and less than 1% of cardiac primary malignancy. Clinical presentation is driven by mass effect related symptoms or hormonal related symptoms from excessive production of catecholamines. Cardiac PGLs can be found in any part of the heart; however, case series data have shown propensity for the left atrium. PGLs of the right atrium are much rarer (6). Approximately 10% of PGLs show malignancy, usually characterized by the presence of metastases (7).

The treatment of metastatic cardiac PGLs is multimodal, including a combination of surgery and/or external beam radiation therapy for the primary tumor and/or systemic treatment. Up to 90% of those tumors express somatostatin receptor (SSTR), making peptide receptor radionuclide therapy (PRRT) with lutetium-177 or yttrium-90-labeled somatostatin receptor ligands a very promising therapeutic avenue. Preliminary data suggest higher response rates and a more favorable toxicity profile for PRRT as compared with ^131^I-MIBG (8).

To our knowledge, this is the only case of metastatic PGL with substantial intracardiac extension treated with personalized ^177^Lu-DOTATATE reported in the literature. Although other cases of cardiac PGLs treated with PRRT have been reported, these included patients with little intracardiac extension or ^90^Y-DOTATATE based PRRT, and did not provide a detailed history, treatment details like injected activity nor follow-up (9).

## Case Presentation

A 47-year-old male, marathon runner, with no medical history developed progressive fatigue following a 65 km run. Over the following months, the patient experienced a few episodes of paroxysmal tachyarrhythmia and his exercise tolerance decreased substantially. After 6 months, he developed a grade 3 dyspnea and persistent tachycardia.

The transthoracic echocardiogram showed a right atrial mass obstructing the tricuspid valve and extending in the atrioventricular groove. Given the degree of valvular obstruction and rapidly progressive symptoms, the patient was referred for urgent open-heart surgery. Only two thirds of the tumor could be safely removed. Post-operative imaging demonstrated a 7.3-cm residual lesion attached to the posterior aspect of the right atrium occupying majority of the cavity and extending in the ventricle, causing mild obstruction of the tricuspid valve. The right systolic function was preserved. Histopathological analysis showed a PGL with a Ki-67 of 30-40%. Genetic testing identified the presence of SDHB gene mutation. Serum and urinary metanephrines and norepinephrines were elevated. Alpha and beta-blocking medication was initiated.

**Figure 1:**
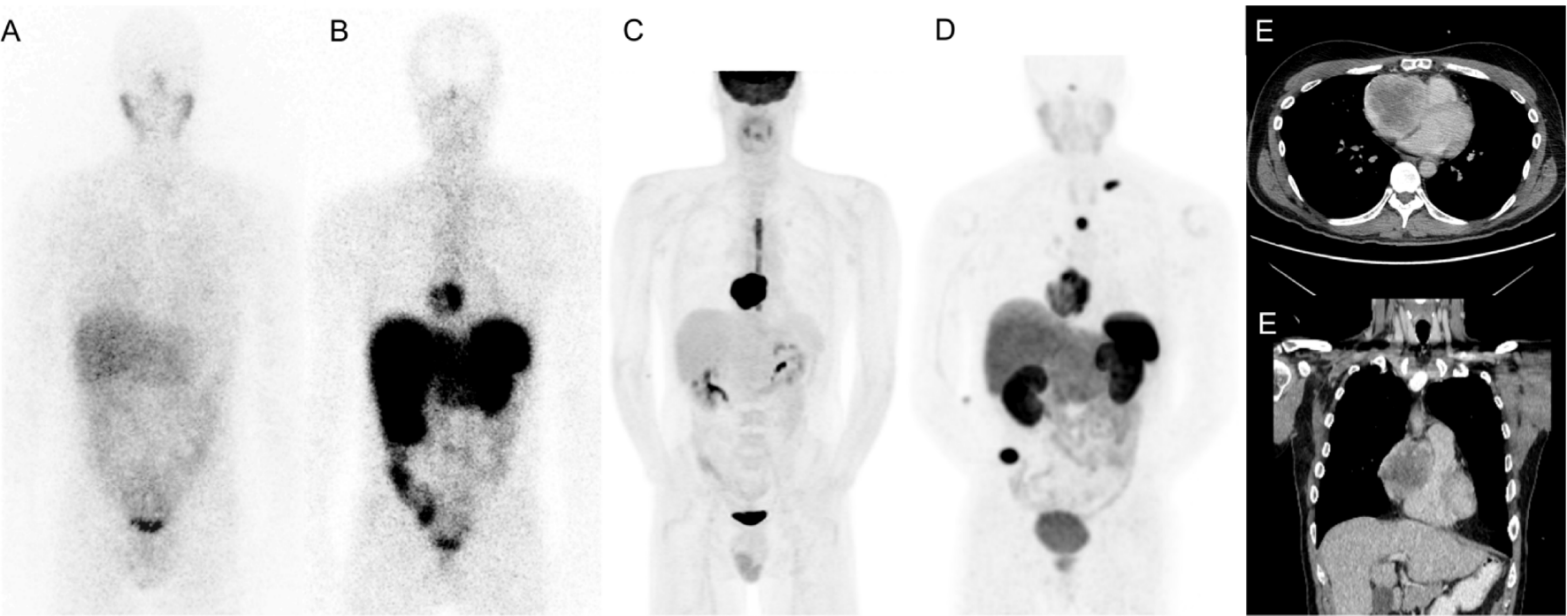
Staging imaging; ^123^I-MIBG scintigraphy **(A)**, ^111^In-Pentetreotide scintigraphy **(B)**, ^18^F-FDG PET/CT **(C)**, ^68^Ga-DOTATATE SPECT-CT PET/CT **(D)**, Contrast-enhanced CT **(E)**.

The ^123^I-MIBG scintigraphy was negative. A ^18^F-FDG PET/CT showed marked hypermetabolism (SUV_max_ 36) and central necrosis of the right intra-auricular mass. ^111^In-pentetreotide SPECT/CT demonstrated a high avidity of the cardiac lesion (**Figure 1**). There was no distant metastasis. Two months later, a ^68^Ga-DOTATATE PET/CT showed intense uptake (SUV_max_ 14) of the cardiac lesion and three new lytic bone lesions consistent with metastases. As these were also new on the accompanying low dose CT scan, their detection was not due to the ^68^Ga-DOTATATE PET-CT superior sensitivity.

Because of the metastatic status, heart transplantation was initially dismissed. Without treatment, the patient symptomatology quickly worsened. The patient was referred for ^177^Lu-DOTATATE treatment as part of our clinical trial of personalized PRRT (NCT02754297). At the time of the first treatment, the primary lesion had progressed to 8,5 x 7,2 x 8,8 cm. The mass was now transgressing the right atrial wall and encasing the right coronary artery over 11 cm. Thus, administration of the highest activity was considered critical in order to maximize the RECIST response (Response Evaluation Criteria in Solid Tumours version 1.1). As a precautionary measure for the theoretical risk of hormonal release, hemodynamic compromise and bleeding, the patient was hospitalized for the first treatment. The protocol included 4 induction cycles every 8-10 weeks administered over 25 minutes in combination with an amino acid infusion. The patient was on alpha and beta blockers. He was also premedicated with dexamethasone and ondansetron for each cycle. Total administered activity was based on a cumulative kidney absorbed dose of 23 Gy and dose was distributed over 4 cycles. The first cycle administered activity was estimated with body surface area and renal function (10). Subsequent ones were estimated with SPECT/CT-based dosimetry with two time points (11). The patient received four cycles of ^177^Lu-DOTATATE and a cumulative activity of 40.7 GBq (1100 mCi), including 12.6 GBq (340 mCi) in the first cycle, well above the empiric activity of 7.4 GBq (200 mCi) per cycle used for GEP-NETs (29.6 GBq total). We estimated that, at 69 Gy, the maximum tumor dose was 37% higher than what would have been delivered during empiric PRRT.

Given the high risk of bleeding, pre-PRRT angioembolization of the main tumor feeder vessels had been initially planned. However, considering the tumor growth rate and the progression of dyspnea, chest pain and tachycardia, the procedure was abandoned and PRRT prioritized. The patient underwent the 4 induction cycles without substantial acute or subacute side effects. He had no hemodynamic toxicity or bleeding. Over the course of his treatments, he had episodes of grade 2 thrombocytopenia and neutropenia, all of which were self-limited (**Table 1**). At the end of the 4 cycles, fatigue, chest pain and dyspnea resolved, and episodes of tachyarrhythmia disappeared. The dosage of metoprolol and prazocin was considerably reduced, decreasing respectively from 125 mg to 50 mg and 2 mg to 1 mg TID. The chromogranin A decreased from 585 to 205 ng/mL and the serum norepinephrine decreased from 104 to 37 nmol/L after 4 cycles.

**Table 1:** Transient hematotoxicity from ^177^Lu-DOTATATE therapy.

Treatment cycle	Platelets (x 10^9^/L)	Neutrophils (x 10^9^/L)	Hemoglobin (g/L)
**1**	211	5.60	120
**2**	170	3.10	134
**3**	157	1.70	131
**4**	74	1.60	122
**3 months after PRRT** **6 months after PRRT**	92 127	1.92 3.00	120 123

**Figure 2:**
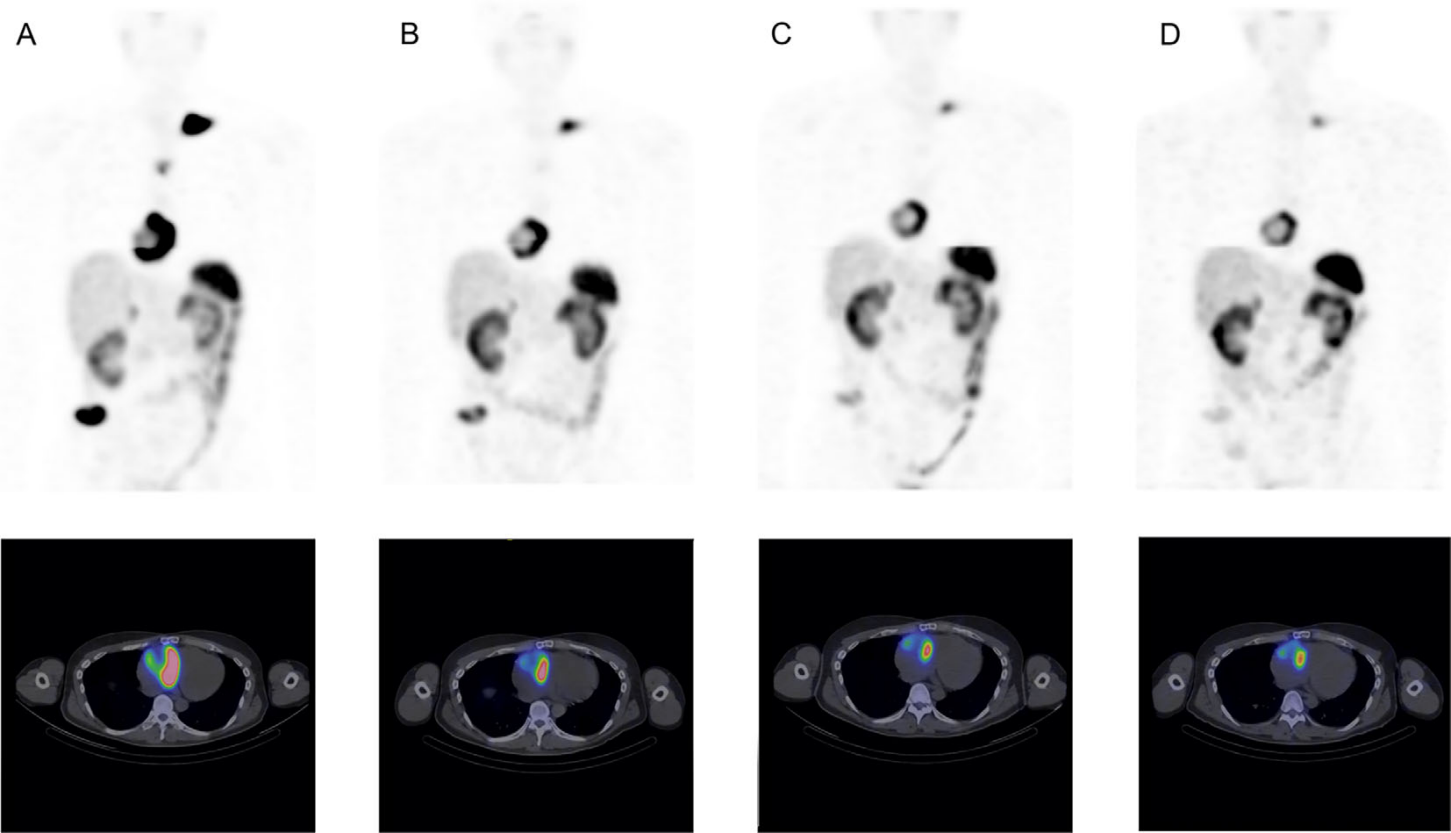
Post-treatment ^117^Lu-DOTATATE maximum intensity projection (MIP) and axial fusion images. Post-treatment images 1 **(A)**, 2 **(B)**, # 3 **(C)** and 4 **(D)**.

**Figure 3:**
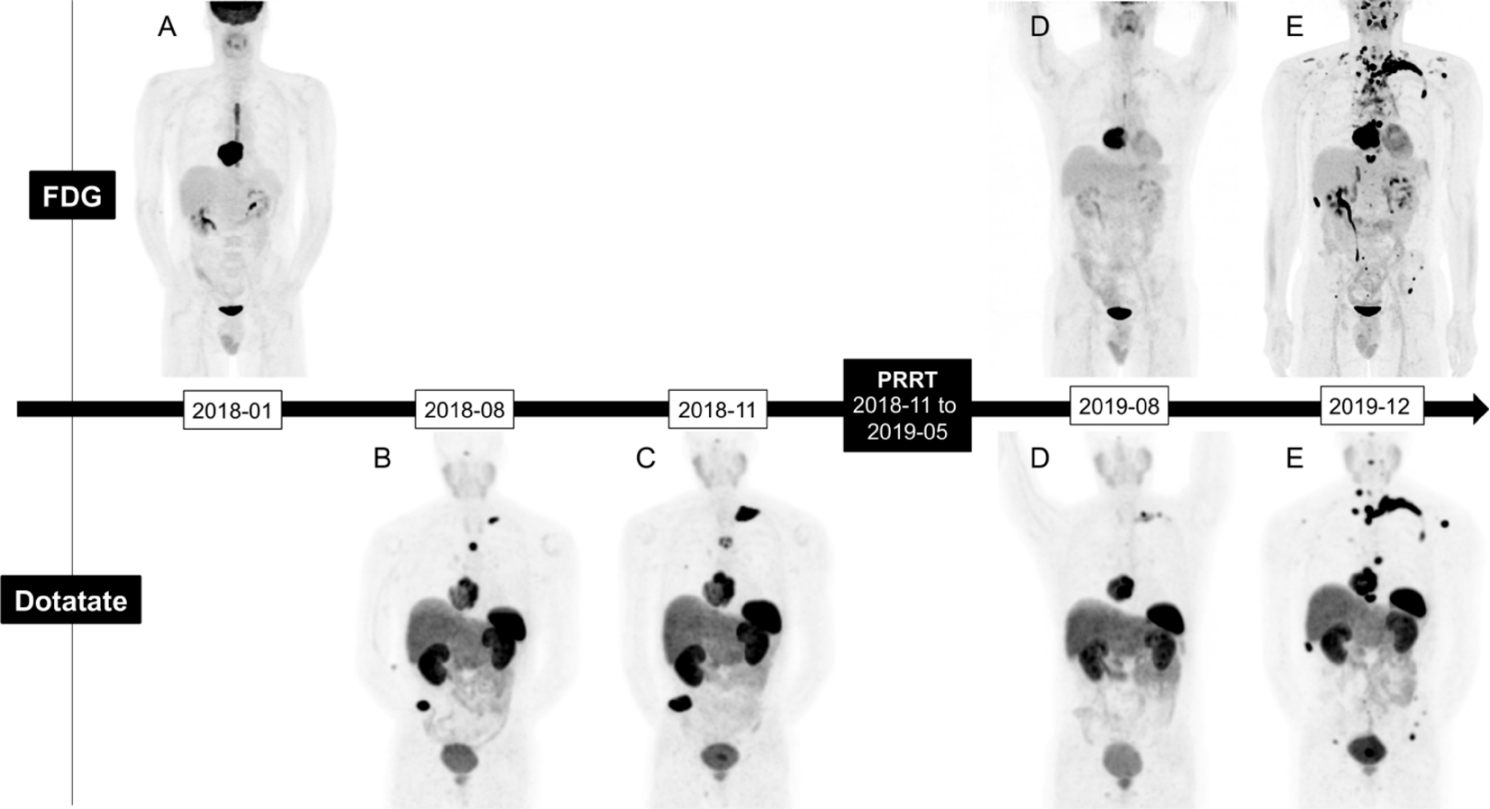
^18^F-FDG and ^68^Ga-DOTATATE PET/CT maximum intensity projection (MIP) imaging evolution; Staging **(A, B)** Pre-treatment **(C)**, 3 months post-treatment **(D)**, 7 months post-Treatment **(E)**.

SPECT/CTs (**Figure 2**) performed following each treatment showed incremental anatomical response. ^68^Ga-DOTATATE PET/CT obtained at three months after the last treatment confirmed the partial response with the main cardiac lesion measuring 5.5 x 4.7 x 6.0 cm compared to 8.5 x 7.2 x 8.8 cm initially (32% reduction in largest dimension). The metabolic tumor volume, as defined by SSTR positive viable tumor identified on ^68^Ga-DOTATATE PET/CT decreased by 62%, from 257 to 99 cc. Unfortunately, the follow-up ^68^Ga-DOTATATE PET/CT (**Figure 3**) at seven months after the last induction cycle showed a significant increase of the primary tumor size (6.8 x 5.6 x 6.8 cm) equivalent to 117 cc, the development of mediastinal adenopathy and diffuse bone metastatic infiltration. The progression-free survival was thus 13 months. Given the rapidity and the extent of recurrence, and subacute bone marrow toxicity, salvage PRRT treatments were not considered.

The patient was evaluated for Sunitinib. The treatment was poorly tolerated and was discontinued after 6 months despite numerous breaks and doses adjustments. Then, he received temozolomide for 6 months until disease progression. Cabozatinib was tried but was discontinued after only one cycle due to persistent thrombocytopenia and poor tolerability. As of May 2021, the patient is receiving best supporting care including external radiation therapy targeted on painful bone metastases.

## Discussion

The main treatment of cardiac PGLs is surgical resection. Complete resection is complex because of their large size, relationship to vital structures, lack of capsule and clear delineation (12). In addition, they have substantial vascularization often originating from the coronary network (6) increasing risk for hemorrhage. A few cases of preoperative embolization have shown a decrease in the risk of bleeding (13). Adrenergic blockade with alpha- and beta-adrenergic antagonists is also recommended to avoid the effect of catecholamine release secondary to surgical manipulation. Partial resection is sometimes considered to reduce symptoms, prevent complications, or promote response to systemic therapy. In our case, a second surgical resection was dismissed because the tumor had become too large and invaded the right atrial wall with important right coronary encasement, making the procedure too risky.

Cytotoxic chemotherapy efficacy for metastatic paraganglioma is not well defined in the literature. A meta-analysis of 50 patients with metastatic paraganglioma treated with chemotherapy regimen comprising cyclophosphamide, vincristine and dacarbazine showed an objective response rate of 41% but only a disease control rate of 55% (combination of complete response, partial response and stable disease according to RECIST 1.1 criteria) (14).

Treatments of metastatic diseases also include β- particles emitting radiopharmaceuticals. Up to 60% of PGL and pheochromocytoma (PC) demonstrate high enough uptake to consider ^131^I-MIBG therapy (15). The benefits of ^131^I-MIBG are poorly defined in the literature due to the lack of prospective studies. The available studies are mostly retrospective and are performed on very heterogeneous populations with variable dose administration protocols. A recent meta-analysis of 243 patients receiving various regimes of ^131^I-MIBG showed a partial response in 27% and disease stability in 52% (16).

^177^Lu-DOTATATE, another medium-energy beta-emitting radiopharmaceutical, is also part of the available therapeutic arsenal. A recent metanalysis including 201 cases of PGL or (PC) treated with ^177^Lu-DOTATATE or ^90^Y-DOTATOC showed an objective response rate of 25% and a disease control rate of 84%. Clinical and biochemical responses were observed in 61 and 64% of patients (17). The median progression-free survival was 37.1 months. Myelotoxicity (grade > 2) as neutropenia occurred in 3%, thrombocytopenia in 9%, and lymphopenia in 11% of cases. Nephrotoxicity (grade > 2), defined as a gradual and usually permanent loss of kidney function resulting in a decreased glomerular filtration rate of 59-30 mL/min, was reported in 4% of cases (17). It should be noted, however, that the patients included in this study were heterogeneous. Neither tumors grade nor the proportion of SDH mutations were provided.

More recently, a retrospective study including 22 patients with PGLs or PCs treated with 3 to 11 cycles of ^177^Lu-DOTATATE, with a median total administered activity of 29.6 GBq (800 mCi) showed a progression-free survival of 21.6 months (18). A subgroup analysis showed a significant increase in progression-free survival for tumors with Ki-67 < 15% and patients treated as first-line therapy. No significant toxicity was noted.

Given the lack of controlled studies or comparative studies between ^131^I-MIBG and ^177^Lu-DOTATATE, it is not possible to determine the superiority of either agent nor can definitive conclusion be drawn from this current case report. The best approach for now is based on tumor characterisation from SSTR and MIBG imaging, opting for the treatment with the highest uptake, considering the toxicity profiles, the characteristics and comorbidities of the patient, and local availability (19). In the case of our patient, ^177^Lu-DOTATATE was the only remaining radiopharmaceutical therapeutic agent in view of the lack of ^131^I-MIBG uptake. A few cases in the literature have shown a benefit in this particular situation (20).

The administration of dexamethasone with PRRT as premedication is controversial for the treatment of PGLs and PCs. Dexamethasone has antiemetic properties, reduces the radiation-induced tumor edema and in this case, reduces the risk of vascular compromise. However, it is thought to increase the risk of a catecholaminergic crisis by increasing the production and release of catecholamines into the circulation, and by its synergistic pharmacodynamic effects, particularly at the level of the vascular endothelium (21). In view of the few cases of catecholaminergic crises reported following treatment of PRRT in patients who have received premedication with dexamethasone, some authors discourage its use (22). For our patient, it was judged that there were more benefits than risks to administer dexamethasone premedication given the location of the PGL and the risk of vascular compromise. Other than a hot flush during the first cycle relieved by temporarily stopping the ^177^Lu-DOTATATE infusion, there were no other reactions related to the release of catecholamines.

A recent phase II registry study from Sistani G and al (23). suggests a potential benefit of increasing the treatment intervals and the number of cycles, each with a lower activity in comparison to phase III NETTER-1 trial (24). Considering the high ki-67 of 30-40% and the rapid recurrence of our patient tumor, it is possible to believe that he could have benefited more from this longer-term, continuous PRRT approach, albeit keeping the cycle intervals short.

## Conclusion

Cardiac PGL is an exceedingly rare tumor and may lead to hemodynamic compromise. Surgical resection, if indicated, is technically challenging and frequently not feasible. This case demonstrates that personalized ^177^Lu-DOTATATE PRRT can be considered as a safe and effective palliative treatment for unresectable MIBG negative tumor which can substantially improve quality of life.

## Patient Perspective

Our patient was very grateful for his treatments. Each treatment, he gained more energy and enjoyed a better quality of life with his family, including his soon to be graduated daughter. He was also grateful to his community for helping him make the trip to treatments.

## Data Availability Statement

The original contributions presented in the study are included in the article/supplementary material. Further inquiries can be directed to the corresponding author.

## Ethics Statement 

The studies involving human participants were reviewed and approved by CHU de Québec - Université Laval research committee. The patients/participants provided their written informed consent to participate in this study. Written informed consent was obtained from the individual(s) for the publication of any potentially identifiable images or data included in this article.

## Author Contributions

AHD and MD are the main contributor of the manuscript. JMB, AB, FA, GA, ET, and FAB acted as a reviewer. FAB is the senior author. All authors contributed to the article and approved the submitted version.

## Conflict of Interest

The authors declare that the research was conducted in the absence of any commercial or financial relationships that could be construed as a potential conflict of interest.

## Abbreviations

FDG, Fluorodeoxyglucose; DOPA, Dihydroxyphenylalanine; MIBG, Metaiodobenzylguanidine; PC, Pheochromocytoma; PGL, Paraganglioma; PRRT, Peptide receptor radionuclide therapy; SDH, Succinate dehydrogenase; SSTR, Somatostatin receptor.
